# Microelectrode Arrays Technology for Brain-on-a-Chip Applications

**DOI:** 10.3390/bios16060305

**Published:** 2026-05-23

**Authors:** Mingda Zhao, Yuxing Zhang, Yibo Wang, Hui Liu, Mingxiao Li, Yang Zhao, Lingqian Zhang, Chengjun Huang

**Affiliations:** 1 Institute of Microelectronics, Chinese Academy of Sciences, Beijing 100029, China; zhaomingda24@ime.ac.cn (M.Z.); zhangyuxing25@ime.ac.cn (Y.Z.); wangyibo25@ime.ac.cn (Y.W.); liuhui2023@ime.ac.cn (H.L.); limingxiao@ime.ac.cn (M.L.); zhaoyang@ime.ac.cn (Y.Z.); 2 University of Chinese Academy of Sciences, Beijing 100049, China; 3 State Key Laboratory of Fabrication Technologies for Integrated Circuits, Beijing 100029, China

**Keywords:** brain-on-a-chip (BOC), microelectrode arrays (MEAs), biosensors, bioelectronics, organoid intelligence

## Abstract

Brain-on-a-chip (BOC) refers to a miniaturized in vitro platform that integrates living neuronal networks on a micro-engineered chip, enabling the simulation of brain functions, neural activities and physiological responses. BOC technology is an advanced evolution of microphysiological systems (MPS) and Lab-on-a-Chip platforms, providing novel paradigms for in vitro modeling and exploring early-stage biocomputing by interfacing living neural networks with engineered electronics. Microelectrode arrays (MEAs) serve as the critical physical interface for bidirectional communication in these systems. In this review, we systematically examine the technological landscape and engineering requirements of MEAs tailored for BOC applications, evaluating them across electrical characteristics, structural properties, and biocompatibility. Two primary classes of current MEA technologies, including planar arrays for 2D neural cultures and 3D flexible arrays for brain organoids, are discussed in detail. We highlight the transition from passive planar electrodes to high-density active CMOS and TFT-based arrays, and detail how 3D flexible MEAs utilize endogenous integration and exogenous wrapping strategies to overcome tissue-mechanics mismatches. Furthermore, the integration of MEAs with microfluidics, optoelectronics, and electrochemical sensors to enable multimodal monitoring is explored. With the advantages of the various MEAs, the application of MEAs for BOC, particularly in biological computing and network plasticity research, is discussed. Finally, future technological developments in scalability bottlenecks, chronic stability, and the incorporation of artificial intelligence for MEAs of BOC are prospected.

## 1. Introduction

Brain-on-a-chip (BOC) is an advanced in vitro microphysiological modeling system that interfaces neural tissue, such as neuronal networks or brain organoids, with microelectrode arrays on a chip, enabling bidirectional communication between living neurons and an electronic circuit for modeling brain function and implementing a brain–computer interface in vitro.

Driven by the increasing demand for energy-efficient computing architectures, BOC technology has progressively evolved from basic neural signal detection to exploring early-stage computational tasks. Crucially, the execution of these biological computations is intrinsically entangled with advanced MEA technology, which serves as the indispensable bidirectional data conduit. For example, high-density MEAs are required to deliver spatiotemporally encoded electrical stimuli and simultaneously extract complex nonlinear responses from brain organoids to achieve pattern recognition [1]. Similarly, closed-loop MEA systems provide the real-time electrical feedback necessary to train cultured neural networks for reinforcement learning tasks, such as playing simulated games [2]. By exploring the preliminary feasibility of biological neural networks as an alternative computing substrate, these milestone studies underscore that sophisticated MEA hardware is the fundamental prerequisite for translating neural activity into computational function. In contrast to conventional implanted brain–computer interfaces, BOC integrates high-density microelectrode arrays for precise bidirectional interaction with cultured neural networks, intersecting with concepts such as organoid intelligence and wetware computing while maintaining its distinct focus on engineered electronic interfacing.

A BOC system typically comprises three essential components: (1) the biological unit, which consists of artificial cultured living neural networks, ranging from primary neurons and induced pluripotent stem cell (iPSC)-derived cultures to three-dimensional brain organoids; (2) the bio-physical (i.e., electrical and/or optical) interface, embodied by microelectrode arrays (MEAs) that enable bidirectional electrical communication with the neural tissue; (3) the supporting microenvironment, provided by microfluidic systems that precisely control nutrient delivery, waste removal, and biochemical cues, often complemented by external signal processing circuitry for real-time data acquisition and stimulation control. Among these, the microelectrode array serves as the critical technology. By capturing extracellular action potentials and local field potentials while enabling closed-loop electrical stimulation, it establishes a stable, high-throughput bidirectional communication hub for non-invasive, label-free, real-time neural interrogation. Its performance, in terms of electrode density, integration level, spatial resolution, signal-to-noise ratio, and long-term stability, directly determines the functional capabilities of the entire BOC platform.

**Figure 1:**
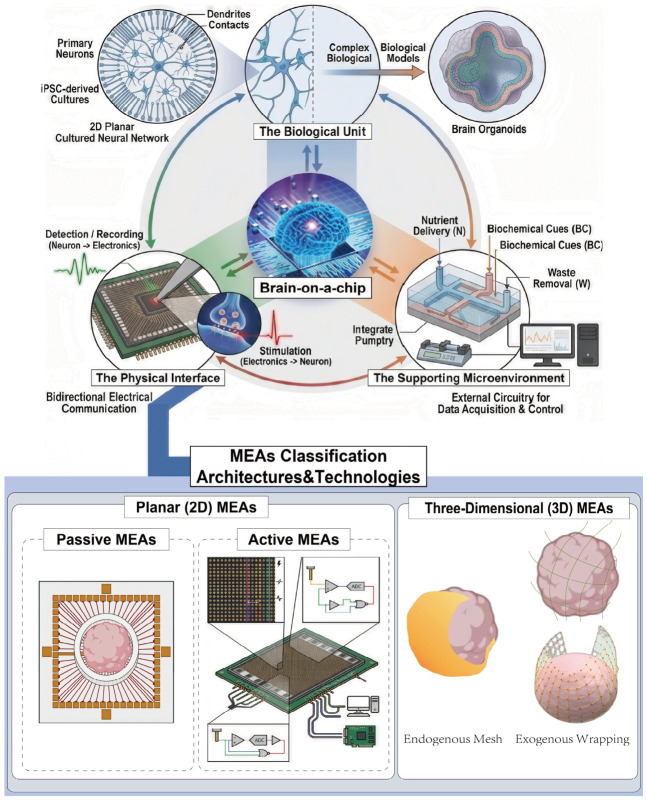
Schematic illustration of the Brain-on-a-chip (BOC) ecosystem and classification of Microelectrode Arrays (MEAs). The upper panel depicts the three pillars of a BOC system: the biological unit, the physical interface, and the supporting microenvironment. The lower panel highlights the technological landscape of physical interfaces, specifically focusing on MEAs: Planar MEAs: Including Passive MEAs, Active 2D MEAs and Three-Dimensional (3D) MEAs: Represented by symbiotic, wrapping, and mesh designs, optimized for spatial topological matching and bioelectrical signal acquisition from in vitro organoids. This figure is an original conceptual illustration prepared by the authors.

Given the central role of microelectrode arrays in BOC systems and the rapid pace of technological innovation in this field, previous reviews have focused on microfluidic architecture [3] and human–machine interaction [4]. This review provides a focused examination of MEA technologies tailored for BOC applications. At first, in this review, we should establish the biological requirements for MEAs derived from neural culture models and propose a corresponding performance evaluation framework. Next, we survey the technological landscape of MEAs, categorizing them into two major classes based on their applications: MEAs for planar cultured neural networks and MEAs for three-dimensional brain organoids. The former commonly include passive electrodes and active electrodes based on complementary metal-oxide-semiconductor (CMOS) or thin-film transistor (TFT) technologies, while the latter mainly include 3D flexible electrodes. Strategies for integrating MEAs with microfluidic culture systems and multimodality enabled by light and chemistry are further discussed, as shown in Figure 1.

## 2. Key Considerations for MEAs for Brain-on-a-Chip Application

In recent years, a series of representative works have emerged in the field of BOC. Notable examples include the “DishBrain” system by Cortical Labs, which demonstrated rapid learning in cultured human brain cells [2]; the proposal of “Organoid Intelligence” by Johns Hopkins University [5]; the “Brain in a Box” system from the University of Illinois for efficient pattern classification [6]; “Brainoware” reported by Indiana University, which integrated brain organoids with electronic components for speech recognition [1]; and the “Neuroplatform” biological processor released by FinalSpark, featuring ultra-low power consumption [7]. These representative studies collectively reveal the fact that the functional realization of the BOC system heavily depends on the precise capture and effective intervention of neural activity by the underlying hardware. Whether it is real-time training of cultured neurons (as in DishBrain), extraction of complex spatiotemporal patterns from three-dimensional brain organoids (as in Brainoware), or the construction of remotely accessible biocomputing platforms (as in Neuroplatform), all require a bio-electrical interface capable of stable, high-throughput, long-term interaction with living neural tissue. To provide an initial overview of the field’s landscape, Table 1 summarizes representative BOC systems, linking their specific technical features with their corresponding biological applications, which will be further discussed in Section 6.

To support the complex applications, MEAs and their peripheral systems are expected to meet strict engineering requirements across multiple dimensions. The first consideration is from the nature of neurophysiological signals. Since neuronal signals include extracellular action potentials (EAPs) with amplitudes of 10–500 μV and local field potentials (LFPs) of 0.5–300 Hz with amplitudes of 0.1–5 mV, MEAs for signal acquisition require a sampling rate of at least 10 kHz and input-referred noise below 5 μVrms (0.1 Hz–10 kHz) to ensure high-fidelity recording [8,9,10]. For micron-sized electrodes (10–50 μm in diameter), an electrochemical impedance of 100 kΩ–1 MΩ at 1 kHz is necessary to maintain a favorable signal-to-noise ratio [11,12]. Second, bidirectional interaction demands not only sensitive recording but also precise stimulation, requiring electrode materials with high charge injection capacity (>0.5–1 mC/cm^2^) and staying within a safe electrochemical window (–0.6 V to +0.8 V vs. Ag/AgCl) to avoid irreversible faradaic reactions. [11,13]. Finally, as the direct interface with living tissue, MEAs must meet strict biocompatibility and long-term stability requirements, supporting continuous culture for weeks to months (typically 4–52 weeks) [14]. This includes compliance with standards such as ISO 10993-5, maintaining cell viability above 90% without significant release of inflammatory cytokines (e.g., IL-6, TNF-α) [15], and ensuring electrochemical stability in cell culture media (37 °C, pH 7.4) with minimal dissolution rates [16,17].

Accordingly, MEA’s performance is typically evaluated across four dimensions: electrical characteristics (impedance, charge injection capacity), recording and stimulation performance (signal-to-noise ratio, long-term cell viability and survival, stimulation safety), structural properties (density, layout, conformability, flexibility), and biocompatibility (cell viability, long-term stability). The subsequent review will assess representative MEA technologies based on these four dimensions. To systematically quantify these structural and electrophysiological demands, a corresponding performance evaluation framework is established, as detailed in Table 2. This framework directly complements the technical criteria discussed above by providing specific metric benchmarks.

## 3. MEAs for Planar Cultured Neural Networks

MEAs for planar cultured neural networks represent the most mature and widely adopted class in BOC systems, serving as the foundation of in vitro electrophysiology for decades. Based on the architecture and signal processing paradigm, they can be further divided into two categories: passive MEAs and active planar MEAs.

### 3.1. Passive MEAs

The core feature of passive MEAs is the most classical and widely used MEA for BOC, as it is fully compatible with the standard Si-based planar microfabrication techniques. This design concept dates back to 1972, when Thomas et al. reported an 18-electrode array for recording extracellular activity from cultured cardiomyocytes, establishing the foundation for modern planar MEAs [18]. Subsequent advances in microfabrication, particularly microelectromechanical system (MEMS) technologies, enabled the production of electrodes with smaller dimensions and higher integration densities, as electrode size and spacing became primarily defined by photolithographic resolution. Representative examples include 64-channel planar passive MEA chips fabricated via MEMS processes, featuring these electrode sizes of 20 × 20 µm^2^ or 40 × 40 µm^2^ and pitches of 140–200 µm for simultaneous extracellular recording [19]. Similarly, Xu et al. reported a planar passive MEA fabricated on a silicon substrate with gold electrodes of 20–50 μm diameter and a pitch of 100 μm, passivated by a SiO_2_/Si_3_N_4_/SiO_2_ sandwich layer, and successfully applied it to extracellular recording of action potentials from cultured cardiomyocytes [20]. MEMS-fabricated passive planar MEAs have also been extended to neurotransmitter detection, as demonstrated by a gold microelectrode array modified with a polymer film for real-time monitoring of quantal exocytosis from single PC12 cells [21]. Over the past decades, passive planar MEAs have been successfully commercialized and widely adopted, with representative systems such as the MEAs2100 series (Multi Channel Systems, Reutlingen, Germany), Maestro Pro (Axion BioSystems, Atlanta, GA, USA), and MED64 (Alpha MED Scientific, Osaka, Japan) providing a widely adopted commercial platform across diverse electrophysiology applications.

Passive MEAs have undergone extensive engineering development, accumulating substantial expertise in electrical interface optimization and electrode layout design. Since neural electrodes form an electrochemical interface with biological tissue, optimizing this interface is essential to ensure stable, long-term neural-electrode interaction with reliable electrical performance. Ideal neural electrodes are typically non-Faradaic, featuring high charge injection capacity and low interfacial impedance. Conventional metallic electrodes (e.g., gold, platinum, platinum/iridium) have been progressively enhanced by incorporating materials such as iridium oxide and titanium nitride, which offer charge injection limits dozens of times higher than platinum [11]. Another effective approach to reduce interfacial impedance is to increase the effective surface area of the electrode site. By increasing the surface roughness using electrodeposition, ion reaction etching, and nano-modification methods [11,22,23], the micro-nano structure integrated electrodes can significantly improve both recording and stimulation fidelity at the electrophysiological interface. In more recent studies, organic conducting polymers and nanomaterials have emerged as highly effective materials for creating biologically active neural interfaces. Organic conducting polymers, typically generated via electrochemical polymerization of conjugated structures with various dopants, support both electronic and ionic charge injection while maintaining low interfacial impedance, high conductivity, and biocompatibility [24,25]. Commonly used conducting polymers include polypyrrole (PPy) [26], polyaniline (PANI) [27], and Poly(3,4-ethylenedioxythiophene) (PEDOT) [28]. Among these, PEDOT has shown particular promise due to its stability in oxygenated, hydrated environments, leading to various proposed modifications. [25,29]. To improve recording and stimulation capabilities, and chronically interact at the cellular level, nanomaterials such as carbon or metallic nanoparticles [30,31], nanofibers [30] and nanotubes [32,33] are also being considered as functional coatings of the electrode. For example, various PEDOT/CNT (Carbon nanotube) composite MEAs have been developed [34,35,36], exhibiting stable electrochemical performance. To achieve optimal signal transduction at the tissue-electrode interface, the selection of sensing materials is critical. Table 3 details the electrochemical properties and biocompatibility of typical electrode materials utilized in current platforms.

**Figure 2:**
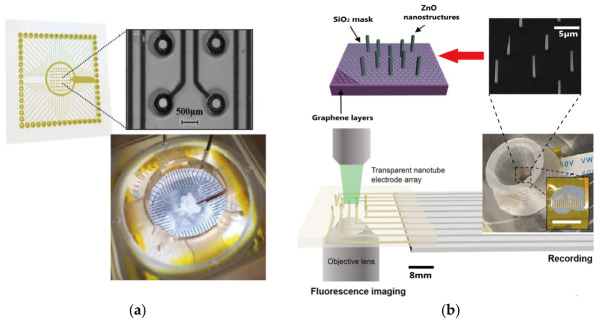
Representative work on passive MEAs: (**a**) Traditional planar electrode array with classic lead-line structure (O’Leary 2022), Reprinted with permission from ref. [39]. Copyright under the terms of the Creative Commons CC BY 4.0 license. (**b**) Two-dimensional planar array modified with transparent vertical nanotubes and ZnO and graphene (Lee 2024), which represents a series of work on increasing the detection area through material modification on a two-dimensional planar electrode array, Reprinted in part under the terms of a Creative Commons CC-BY 4.0 license from ref. [41], Copyright © 2024, The Author(s).

Aiming to overcome the limitations of conventional electrodes in signal coupling efficiency and spatial resolution while meeting the demands of BOC models, apart from the classic two-dimensional planar array electrode, similar to the OpenMEA structure proposed by O’Leary et al. [39], as shown in Figure 2a, diverse strategies in electrode structure and layout design have also been proposed. Three-dimensional topological structures, such as gold nanomushrooms [40], vertical nanopillars [41], as shown in Figure 2b, and transparent vertical nanotubes [42] have been developed to achieve tight membrane wrapping or direct intracellular penetration, enabling high-amplitude intracellular recordings. To enhance interface sealing, strategies including microneedle electroporation arrays [43], silicon-based microwell arrays for GΩ-level sealing [44], and microfluidic negative-pressure adsorption [45,46] have achieved patch-clamp-like recording quality while maintaining planar substrate compatibility. Furthermore, customized electrode layouts have emerged in response to increasingly sophisticated BOC models. Beyond standard orthogonal grids, hexagonal packing arrangements offer isotropic intercellular connection information with higher spatial utilization [47]. For multi-brain-region co-culture networks, stepped layouts with high-density centers and sparse peripheries, or cross-region detection zones integrated with microfluidic channels, have been designed to accurately track signal projections from nerve bundles [48,49,50].

Recent BOC applications have also been built upon passive MEAs, exemplified by FinalSpark’s Neuroplatform [7]. This system integrates passive MEAs comprising four groups of eight-electrode arrays with a microfluidic system, achieving a 30 kHz sampling rate via an Intan RHS32 controller. It supports remote access for worldwide researchers to conduct long-term experiments on living biological neurons. However, conventional passive MEAs face inherent limitations. The passive routing scheme results in a trade-off between electrode count and signal integrity: as the number of electrodes increases, the length of interconnecting traces grows, leading to increased parasitic capacitance, crosstalk, and susceptibility to electromagnetic interference. Consequently, the practical electrode density in conventional systems is constrained to approximately 10^2^–10^3^ electrodes per chip.

### 3.2. Active MEAs

Since passive MEAs lack active switching devices within the array area, requiring each recording site to be individually fanned out to a peripheral contact pad via a dedicated physical trace, they face the insurmountable physical limitation in improving electrode density and spatial resolution. To enable high-density addressing of thousands of electrodes within millimeter- to centimeter-scale chips, microelectronics engineers monolithically integrate sensing electrodes with front-end circuits on a single chip, giving rise to active MEAs. A key advantage of active electrodes is the on-chip integration of an amplifier, which greatly enhances the Signal-to-Noise Ratio (SNR) of weak electrophysiological signals buried in background noise. By incorporating transistor switches or amplification circuits in situ at each recording site, active MEAs employ time-division multiplexing to transmit multiple channels of electrophysiological signals over a single physical lead, fundamentally eliminating the wiring-induced constraints on electrode density.

#### 3.2.1. CMOS-Based Active MEAs

Commercial high-density active MEA platforms, such as MaxWell Biosystems’ MaxOne and MaxTwo, have been developed based on Si CMOS-MEMS integration and have gained widespread recognition in brain-on-chip research. MaxOne integrates 26,400 platinum electrodes (17.5 μm pitch, 3265 electrodes/mm^2^) with 1024 parallel recording channels, a 20 kHz sampling rate, 2.4 μVrms input-referred noise, and 32 stimulation units, enabling subcellular-resolution extracellular recording and control. MaxTwo extends this capability to multi-well plates (6- or 24-well), with built-in temperature and CO_2_ control for long-term, high-throughput parallel experiments. Unlike traditional passive MEAs, both MaxOne and MaxTwo are built upon a CMOS active MEA architecture, where amplifiers, filters, analog-to-digital converters (ADCs), and stimulation circuits are monolithically integrated into the same chip substrate under the platinum electrode array, enabling each electrode to be addressable and to locally process the acquired signal. This design allows the signal conditioning circuit to be as close as possible to the sensing site, thus greatly shortening the length of the high-impedance lead, effectively suppressing external electromagnetic interference and lead parasitic capacitance, and achieving ultra-low noise recording and high spatial and temporal resolution parallel acquisition.

**Figure 3:**
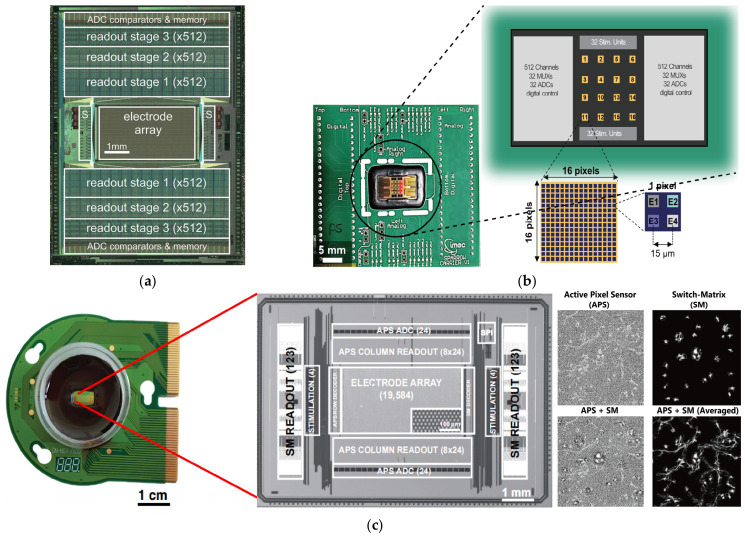
Representative technical solutions for CMOS-MEAs: (**a**) Switch Matrix CMOS-MEAs (Ballini 2014 & Müller 2015), Reprinted with permission from refs. [44,45]. Copyright Royal society of chemistry 2015 under a Creative Commons Attribution 3.0 Unported License. (**b**) APS CMOS-MEAs (Miccoli 2019), Reprinted with permission from ref. [48].Copyright© 2019 Miccoli, Lopez, Goikoetxea, Putzeys, Sekeri, Krylychkina, Chang, Firrincieli, Andrei, Reumers and Braeken. Under the terms of the Creative Commons Attribution License (CC BY). (**c**) Dual-Mode CMOS-MEAs (Yuan 2020), which represents the work of CMOS-MEAs integrating SM & APS integrated on-chip, Reprinted with permission from ref. [51], Copyright under the terms of the Creative Commons Attribution License (CC BY).

Both MaxOne and MaxTwo systems are built upon the switch-matrix architecture pioneered by Hierlemann’s group at ETH Zurich. This architecture densely lays out a static random-access memory (SRAM) network beneath the microelectrodes, while placing low-noise amplifiers (LNAs) and ADCs at the chip periphery. This architecture has gone through continuous iterations: Frey et al. reported an 11016-electrode chip (126 channels, 17.8 μm pitch, 2.4 μVrms noise, 135 mW), which enabled fine tracking of single-axon action potential propagation over >1.5 mm, revealing conduction velocity heterogeneity (0.4–0.9 m/s) in unmyelinated axons [43]; Ballini M et al. increased electrodes to 26,400 and channels to 1024 (17.5 μm pitch, 3265/mm^2^, 75 mW), with platinum black modification reducing interfacial impedance [44,45], as shown in Figure 3a. Vijay V et al. expanded to 59,760 electrodes with 32-channel impedance measurement [46], and Yuan et al. integrated 59,760 electrodes, 4096 channels, and large-scale concurrent stimulation [47].

Unlike the switch matrix, which relies on external routing for channel gating, the active pixel sensor (APS) architecture integrates a transistor-level amplifier directly beneath each microelectrode, enabling full-array simultaneous recording. Berdondini et al. first reported the application of this architecture in CMOS-MEAs. They demonstrated a 4096-electrode CMOS-MEAs (42 μm pitch, 7.8 kHz, 40 dB gain) with on-pixel auto-zeroing for DC drift suppression [52]. This platform supported three-month continuous recording of hiPSC-derived neural networks in vitro [53]. Subsequent improvements include Sony’s low-noise APS (4.8 μVrms, scalable to 12 μm pitch and 6912 channels) [54] and IMEC’s multimodal APS (16,384 TiN electrodes, 15 μm pitch, six operating modes, 1024 parallel channels) [48], as shown in Figure 3b. Although the APS architecture has significant advantages in full-array synchronous recording and long-term stability, it suffers from higher single-channel noise (4.8–10.9 μVrms) than switch-matrix designs due to limited pixel area for 1/f noise filtering capacitors.

Beyond the switch-matrix and APS architectures, researchers have also explored new readout architectures to break through the scalability bottleneck. The subarray multiplexing (SAM) architecture integrates 24,320 electrodes with 380 readout channels, using time-division multiplexing to allow each analog front-end channel to sequentially record from multiple electrodes, thereby easing the trade-off between routing complexity and channel count [55]. Dual-mode architectures combine complementary readout strategies on a single chip. Yuan et al. integrated APS and switch-matrix modes, enabling on-demand switching: APS mode supports full-array synchronous recording of 19,584 electrodes (11.6 kHz, 10.9 μVrms) for high-throughput network-level recording, while switch-matrix mode provides 246 high-SNR channels (24.4 kHz, 3.1 μVrms) for weak signal detection [51,56], as shown in Figure 3c. Building on this, Xu et al. extended the dual-mode concept to simultaneous chemical-electrical recording. The chip integrates 24,576 electrodes (10 μm diameter, 18 μm pitch) with subarray multiplexing, achieving both spontaneous spike recording from primary neurons and full-array electrochemical imaging of dopamine with a detection limit down to tens of nM [57]. Another innovative direction monolithically integrates CMOS circuits with vertical nanostructures. Abbott reported that a chip incorporates over 1000 recording sites, each containing nine vertical nanoelectrodes (100 nm tip diameter, 3 μm height), along with on-chip recording amplifiers, electroporation stimulators, and digital memory [58]. The natural wrapping of the nanopillars by the cell membrane was used to form a tight seal, and intracellular contact was achieved by applying a brief electroporation pulse. Intracellular action potentials were successfully obtained from 235 cardiomyocytes simultaneously, with amplitude jumping from 50–100 μV (extracellular) to 2–5 mV (intracellular). The SNR increased by more than two orders of magnitude compared with the extracellular recording. In order to more clearly display and compare the working principles and characteristics of the two active MEA structures, SM and APS, and to better expand and summarize more types of active CMOS-MEAs, Table 4 and Table 5 are shown from the unit circuit processing principle and performance level, respectively. It is worth mentioning that Table 5 lists a relatively comprehensive variety of CMOS-MEAs and corresponding data indicators representing work for researchers’ reference.

#### 3.2.2. TFT-Based Active MEAs

Although CMOS-based active MEAs achieve exceptional spatiotemporal resolution, their opaque substrate prevents compatibility with microscopic optical observation and optogenetics study, and their mask-size limitation leads to high manufacturing costs. These drawbacks have driven the development of transparent and scalable thin-film transistor (TFT)-based alternatives. Shaik et al. first reported a fully transparent integrated chip based on a hybrid passive-active matrix architecture, integrating 30,000 indium tin oxide (ITO) transparent electrodes over an ultra-large area of 15.6 mm^2^ (50–100 μm pitch), achieving full compatibility with inverted microscopes [59]. The platform employs a switch-matrix architecture with on-chip TFT switches, enabling dynamic multiplexing of 30,000 electrodes using limited readout channels. Hu et al. further expanded the sensing area to 15 × 15 mm^2^ with 22,500 ITO electrodes (100 μm pitch), and integrated two-dimensional impedance spectroscopy (100 Hz–1 MHz) into the TFT electrode array, enabling real-time cell attachment monitoring and high-resolution impedance mapping [60]. In addition, the “NeuroCam” system by the Sheng’s Group realizes active multiplexing and integration of up to 4096 parallel channels based on flexible TFT technology. It constructs a high-density electrode array on a 15 μm ultra-thin polymer substrate, achieving conformal attachment to curved tissue while maintaining high spatial sampling density [61]. These works established TFT-based active MEAs as a large-area, fully transparent, high-density, and multifunctional on-chip electrode platform, providing key technical support for brain-on-chip interfaces compatible with optical imaging. Table 6 shows some details of these representative TFT-based active MEAs.

### 3.3. Quantitative Engineering Trade-Offs: CMOS vs. TFT MEAs

While TFT-MEAs offer unparalleled large-area optical transparency and intrinsic flexibility, their widespread adoption in high-resolution BOC platforms remains constrained by fundamental semiconductor physics. To systematically elucidate these constraints and explain the enduring dominance of silicon technologies, Table 7 provides a quantitative engineering comparison between traditional rigid CMOS and flexible TFT architectures.

As detailed in Table 7, traditional rigid CMOS MEAs—leveraging highly mature silicon foundry nodes—achieve ultra-low dynamic noise floors (typically <5 μV rms) and support massive channel densities often exceeding 10,000 channels/mm^2^ [44]. In stark contrast, current TFT architectures are physically limited by the lower carrier mobility inherent to amorphous oxides (e.g., IGZO) or organic semiconductors. Consequently, emerging TFT-MEAs typically suffer from higher baseline noise (e.g., 30–60 μV rms) and constrained switching speeds, which limit the maximum sampling rate per multiplexed channel to below 10 kHz [61]. This presents a critical bottleneck for resolving high-frequency fast-spiking action potentials.

Furthermore, this fundamental mobility limitation directly impacts power efficiency and scalability. To achieve the necessary transconductance for neural signal amplification, TFTs require comparatively higher driving voltages than highly scaled sub-micron CMOS transistors. This leads to higher localized power consumption and undesirable Joule heating, which is highly detrimental to temperature-sensitive long-term biological cultures. From a fabrication perspective, while low-temperature processing allows TFTs to be directly deposited onto substrate polymers, they cannot yet replicate the extreme lithographic miniaturization achieved by standard CMOS foundries. Therefore, until significant breakthroughs in semiconductor mobility and power efficiency occur, TFT-MEAs will remain a specialized solution for macro-scale compliant interfacing, rather than replacing CMOS as the universal gold standard for single-cell resolution BOC platforms.

## 4. MEAs for Three-Dimensional Brain Organoids

### 4.1. 3D MEAs Material Selection

As brain-on-chip targets shift from planar neural networks to 3D neural cultures and brain organoids, traditional 2D planar MEAs face fundamental spatial probing limitations. Classical 3D arrays, such as metal microwire electrodes, silicon-based Michigan arrays, and Utah arrays, were originally designed for in vivo implantation, offering high-density recording sites on rigid probes. However, the rigidity of silicon (Young’s modulus ~190 GPa) severely mismatches that of neural tissue (~2.5 kPa), causing tissue damage and foreign-body immune responses. [62,63]. This makes them unsuitable for long-term integration with delicate brain organoids. Therefore, a growing number of researchers put their efforts into developing neural electrodes based on soft and flexible materials [17,64]. Polymers such as polyimide (PI), polydimethylsiloxane (PDMS), parylene-C, and SU-8 have attracted extensive attention because of their excellent mechanical flexibility, microfabrication compatibility and biocompatibility [65,66].

Although low-modulus materials are often desirable, this does not mean that soft materials can be applied to all practical situations. For brain organoids that undergo continuous and dynamic 3D volumetric changes during incubation, in-plane tensile compliance becomes even more critical. To address this, Zhenan Bao’s group developed “intrinsically stretchable electronics” through molecular-level chemical design [67,68]. By incorporating stretchable materials such as PDMS and thermoplastic polyurethane (TPU) into the substrate, they achieved a modulus matching brain tissue (tens of kPa) with >100% non-destructive stretchability. At the conductive level, nanoscale blending of PEDOT: PSS with plasticizers overcame microcrack formation in traditional metal films, yielding tissue-biomimetic conductive hydrogels with high conductivity [69,70]. These materials reduce 1 kHz impedance below 50 kΩ and maintain stable SNR during organoid growth. The tissue-biomimetic fibers, such as NeuroString, further developed by this team, can not only penetrate deep into dynamically moving biological tissues for high-fidelity electrophysiological extraction but also integrate in situ electrochemical sensing capabilities. Table 8 systematically summarizes the performance parameters of common flexible materials currently used in BOC applications.

Another recently reported approach to address issues of mechanical mismatch is through shape optimization of the electrodes. The bending stiffness, which scales cubically with thickness, can be dramatically reduced by decreasing device dimensions. It is demonstrated that the electrodes with microscale fiber structures [77,78] and meshed shape [79,80] composed of materials with Young’s modulus in the GPa range have achieved less impact on the surrounding neural tissues. The reduced micron-scale dimensions of neural electrode sites can also permit isolation of individual action potentials and improved tissue compatibility.

It is worth noting that, building on these advances in mechanical compliance, 3D flexible microelectrode arrays (MEAs) have undergone a paradigm shift in how they interface with brain organoids. To overcome the limitations of planar electrodes and avoid chronic damage from rigid probes, current 3D flexible arrays physically interact with organoids in two ways: endogenous integration and exogenous interfacing (wrapping and physical capture).

### 4.2. 3D MEAs for Endogenous Integration

**Figure 4:**
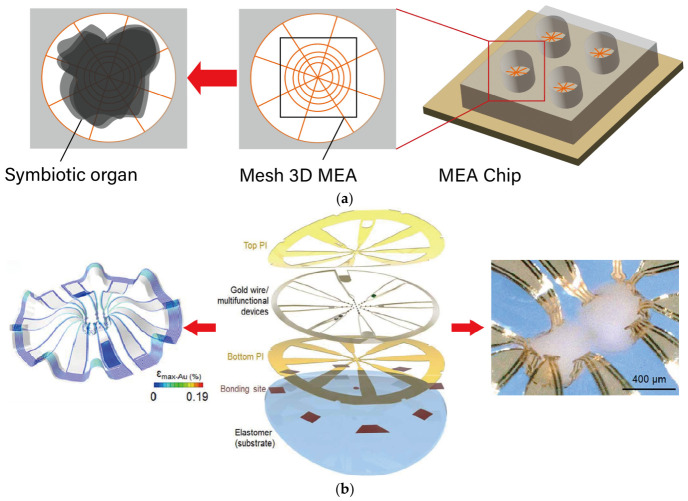
Representative work on 3D-MEAs: (**a**) Mesh MEAs (McDonald 2023), which integrates a symbiotic structure that realizes mesh wrapping as the organ grows, redrawn based on the inspiration from ref. [81]. (**b**) shows a typical structure with a 3D wrapping structure (Park 2021), Reprinted with permission from ref. [92]. Copyright @ 2021 under a Creative Commons Attribution NonCommercial License 4.0 (CC BY-NC).

Endogenous integration flexible arrays leverage high structural porosity and tissue-level flexibility to merge seamlessly with organoids during early development. By closely matching the mechanical stiffness of neural tissues and utilizing subcellular feature sizes, these MEAs minimize the foreign body response and mechanical mismatch. As the organoids undergo organogenesis, cells naturally proliferate and migrate through the highly porous networks, enabling long-term, non-destructive embedded recordings without forming necrotic cores [81]. The conceptual foundation for this approach is comprehensively outlined in the “mesh nanoelectronics” framework by Lieber’s group [82]. Building upon this ultrastructural foundation, Jia Liu’s group experimentally demonstrated the first “cyborg organoids” [83]. In this paradigm, sensor networks with >80% porosity are introduced into stem cell cultures, allowing tissues to naturally envelop the electronics as organoids develop. To optimize this design for improved nutrient diffusion and morphology preservation, McDonald et al. developed a suspended hammock-like mesh array on an ultrathin polyimide substrate. This open architecture gently supports the organoids away from rigid surfaces, allowing unimpeded gas exchange and cellular growth, thereby achieving continuous, high-quality recording from neural organoids for over one year [81], as shown in Figure 4a. To further address the volumetric expansion of growing tissues, Le Floch et al. designed stretchable mesh nanoelectronics that dynamically accommodate tissue growth. This biomimetic tissue reconfiguration enables longitudinal, single-cell-level electrophysiological tracking within developing brain organoids, capturing precise developmental trajectories and individual action potentials for up to six months [78]. Expanding on these topological innovations, Tian’s group developed biomimetic sponge-like 3D macroporous scaffolds. These frameworks provide extensive internal surface areas that actively guide the self-organization of neural stem cells while simultaneously mapping their deep-tissue connectivity [84].

Beyond structural porosity, optimizing the biomimetic mechanical properties of the arrays has become a key focus for endogenous integration. Recent advancements have transitioned from traditional polymers to ultra-soft elastomers and conductive hydrogels to better match the modulus of brain tissue. For example, highly stretchable meshes constructed from the poly (styrene-ethylene-butylene-styrene) (SEBS) encapsulated with PEDOT: PSS conductive hydrogels have been developed [85]. These ultra-soft interfaces act as artificial bio-scaffolds during the embryoid body stage, guiding tissue morphogenesis while eliminating the foreign body response, thereby enabling both high-fidelity recording and stable electrical stimulation for over three months. Furthermore, other advanced, highly transparent carbon-based materials have been engineered into flexible 3D meshes. Most recently, Gao et al. engineered ultrathin graphene into tissue-like 3D flexible meshes [86]. This highly biocompatible, transparent scaffold natively integrates and co-develops with growing 3D microtissues, converging multifunctional tracking capabilities. Reserving the optical window enables seamless, simultaneous high-density electrical mapping and high-resolution live-cell optical imaging (e.g., calcium transients) without obstructing the microscopic field of view, thereby providing a powerful multimodal paradigm for advanced organoid research.

### 4.3. 3D MEAs for Exogenous Interfacing

Exogenous interfacing (such as wrapping and physical capture, etc.) arrays primarily rely on 3D thin-film self-assembly to form cage-like or shell-like structures that envelop pre-formed, mature brain organoids from the outside or gently penetrate the tissue. This approach is highly advantageous for on-demand interfacing without disrupting early, delicate developmental stages. The 3D assembly typically relies on compressive buckling mechanics or environmentally responsive actuation. Rogers’ group utilized the compressive stress from pre-stretched elastomers to drive 2D thin-film bending [79]; their “Kirigami” electronics with spiral interlocking patterns self-fold into basket-like structures to perfectly wrap human cortical organoids, successfully recording spontaneous activity for up to 179 days [80]. For environmentally responsive designs, Huang et al. developed a bilayer self-folding shell MEAs [87], and Lacour’s team created the e-Flower platform using the swelling properties of hydrogels to drive the folding of polyimide electrodes [88]. These exogenous wrapping arrays have all achieved omnidirectional electrophysiological recordings on brain spheroids hundreds of micrometers in diameter, with spike counts (i.e., extracellularly recorded action potentials) and the signal-to-noise ratio (SNR) significantly outperforming traditional 2D planar electrodes.

Building upon these achievements in omnidirectional surface mapping, the frontier of modern exogenous MEAs is rapidly advancing towards seamless multimodal monitoring and deep-tissue interrogation. A notable breakthrough in this domain is the engineering of optically transparent, shell-like MEAs constructed from self-folding polymer leaflets. These visually unobtrusive cages provide 3D omnidirectional electrical coverage while completely preserving the optical window, thereby enabling researchers to conduct simultaneous, artifact-free high-resolution calcium imaging and electrophysiological tracking. Furthermore, to transcend the inherent limitations of conventional conformal shells and deeply interrogate the intricate internal architectures of complex tissues, researchers have pioneered true 3D physical geometries. For instance, Phouphetlinthong et al. ingeniously designed protruding cantilever MEAs capable of warping and rising vertically from the substrate; this active protrusion enables the microelectrodes to gently penetrate the outer layers and directly monitor the deep-tissue inner electrical activity of cerebral organoids [89].

Complementing these multi-modal and penetrating strategies, the field is also evolving to handle complex physical capture and multi-regional tissue models. Soscia et al. successfully integrated flexible MEAs with 3D microfluidic channels to non-destructively trap and dynamically perfuse 3D neural cultures, ensuring long-term viability and stable electrode-tissue interfaces [90]. Moreover, as the focus shifts from isolated organoids toward complex, multi-regional ‘assembloids’, researchers have drawn structural inspiration from early self-folding polymeric containers [91], and consequently, flexible arrays with customized ‘3D pockets’ or soft sandwich-like architectures have emerged [92], as shown in Figure 4b. These adaptive 3D microstructures conformally envelop the highly irregular boundary regions of fused tissues. Ultimately, this tailored physical confinement provides unprecedented access to elucidate long-range functional connectivity and complex spatiotemporal signal propagation across distinct, interacting brain regions.

In summary, MEAs for three-dimensional brain organoids have abandoned the “rigid penetration” paradigm, advancing toward “flexible wrapping” and “endogenous integration” strategies tailored for in vitro organoids. This dual innovation in material mechanics and 3D topology provides the critical physical interface paradigm for next-generation BOC models.

### 4.4. Trade-Offs in 3D Interfacing Strategies

As 3D MEAs advance towards tissue-conformal designs, a critical trade-off emerges between endogenous integration (e.g., mesh nanoelectronics) and exogenous interfacing (e.g., self-folding shells and kirigami structures). Endogenous mesh arrays excel in establishing seamless, true 3D spatial mapping and grow alongside the organoid, which effectively minimizes the foreign body response. However, this strategy requires invasive intervention during the early embryoid body stage and permanently entwines the electronics with the tissue, making post-culture electrode detachment or tissue sectioning virtually impossible without destroying the sample. In contrast, exogenous wrapping MEAs allow for on-demand interfacing with mature, pre-formed organoids and enable easier detachment. The trade-off, however, is that self-folding structures typically confine electrophysiological mapping to the organoid’s cortical surface, often missing critical deep-tissue dynamics unless combined with penetrating architectures.

### 4.5. Long-Term Stability and Encapsulation Strategies

Although 3D flexible MEAs provide excellent mechanical compliance for tissue-conformal integration, their chronic stability—a critical requirement for BOC cultures lasting weeks to months—remains a severe bottleneck. In warm, aqueous cell culture media, conventional conductive polymers used for recording sites (such as PEDOT: PSS) are highly susceptible to swelling, hydrolytic degradation, and delamination from the underlying metal traces. Concurrently, ultra-thin polymer substrates (e.g., polyimide or parylene) may develop microcracks under continuous mechanical pulsation or volume expansion of the organoids, leading to fluid ingress, increased leakage currents, and severe impedance drift.

To specifically address the chronic instability of conductive polymers like PEDOT: PSS, researchers have developed a series of advanced material engineering strategies. First, chemical modifications such as acid treatments (e.g., post-treatment with sulfuric acid) or secondary doping are widely utilized to enhance the crystallinity and wash-resistance of the PEDOT networks, significantly preventing dissolution in aqueous media [93]. Second, robust electrode surface modifications employing potent crosslinking agents (such as GOPS) or adhesion promoters are applied to create strong covalent bonds between the conductive polymer and the underlying substrate, effectively resisting delamination caused by fluid shear stress [94]. Furthermore, incorporating conductive hydrogel materials offers a biomimetic solution; these hydrogels not only match the mechanical modulus of brain tissue to minimize interfacial stress but also provide a stable, highly hydrated 3D microenvironment that shields the conductive networks from severe structural degradation [95].

Beyond optimizing the electrode site coatings, protecting the overall flexible architecture is equally critical. Advanced encapsulation strategies are actively being developed to combat fluid ingress. Atomic layer deposition (ALD) of nanoscale hermetic dielectric coatings (such as Al_2_O_3_ or HfO_2_) has shown immense promise in creating pinhole-free barriers against ion penetration [96]. Furthermore, recent breakthroughs in intrinsically stretchable, self-healing elastomers and highly stable carbon-based nanocomposites [97,98] provide robust pathways to maintain stable electrochemical impedances and prevent encapsulation delamination over extended longitudinal monitoring periods.

## 5. MEAs Integration with Multi-Functional Modules

### 5.1. MEAs Integration with Microfluidics and Flexible Interfaces

Constructing adaptive conformal interfaces, BOC usually needs to be carried out under a sterile environment with as little human operation as possible, and the integration of microfluidics has greatly improved the reliability of the system in aspects such as drug administration and measurement. Voitiuk’s group reported a microfluidic external circuit combined with a microfluidic mechanical pump [99] and an on-chip tissue probe [100] to achieve signal extraction. To further bridge the gap between rigid fluidic chambers and soft neural tissues, integrating flexible interfaces has become crucial. For example, Soscia et al. successfully embedded a highly flexible, polyimide-based MEA within a 3D microfluidic device [90]. This hybrid architecture not only facilitates the automated, localized perfusion of pharmacological agents but also allows the flexible electrode array to softly conform to 3D neural cultures. This synergy ensures stable, shear-stress-free electrophysiological monitoring over extended periods without inducing tissue damage.

### 5.2. MEAs Integration with Optoelectronic Modules

Electrophysiology offers high temporal resolution, but cannot distinguish neuron types, while optical imaging provides single-cell spatial targetability. The “optrode” bridges these modalities for closed-loop neural modulation. To overcome the visual blind spots caused by traditional opaque metal electrodes under optical/fluorescence microscopy, transparent, flexible MEAs integrated with graphene and ITO composites [101] have been developed. This light-transmitting architecture allows for the interference-free, simultaneous extraction of single-cell fluorescent calcium imaging and population action potentials. Meanwhile, for in vitro BOC platforms, researchers have introduced optical stimulation modules in situ within neural networks cultured on MEAs [102]. By employing closed-loop optoelectronic approaches for high-precision spatiotemporal intervention, this integration technology has demonstrated tremendous potential in brain-inspired computing and neural plasticity research.

### 5.3. MEAs Integration with Electrochemical Sensors

Neural signal transmission relies not only on electrical activity but also on the dynamic release of neurochemical transmitters such as dopamine and glutamate. A singular MEA lacking chemical sensing capability cannot fully capture the chemical communication within neural networks. This has driven the development of electrophysiological-electrochemical multimodal integrated systems. By employing targeted surface modification strategies—such as utilizing highly conductive nanocomposites, for example, PEDOT: PSS, platinum nano particles (PtNPs) at electrophysiological sites to lower interfacial impedance, and functionalizing chemical sites with specific enzymes or carbon nanomaterials (e.g., CNTs/rGO) to enhance sensitivity—simultaneous electrical detection and electrochemical sensing have been achieved on a single in vitro MEA chip [103,104]. This multimodal integration scheme effectively suppresses the electrode crosstalk between high-frequency voltage signals and weak current signals within micro/nanoscale spaces. Consequently, it successfully enables the real-time, bimodal synchronized mapping of local field potentials (LFPs), spikes, and neurotransmitter (such as glutamate or dopamine (DA)) concentration fluctuations across cultured neural networks and brain slices.

### 5.4. MEAs Integration with 3D Scaffolds

For 3D brain organoids, planar MEAs suffer from inadequate electrode-cell coupling. Beyond buckling-based assembly, strain-induced self-rolling of thin films has emerged as a powerful strategy to transform planar electronics into 3D micro-cylindrical scaffolds. For example, Kalmykov et al. developed the ‘organ-on-a-chip’ platform, where sensor arrays self-assemble into 3D shell structures to snugly wrap human brain spheroids [105]. These 3D scaffolds facilitate high-density, omnidirectional electrical interrogation across the curved surface of the organoid, providing a robust hardware paradigm for tracking developmental electrophysiology and drug responses in a stable 3D environment. Table 9 provides the typical structure and features of these MEAs’ integration.

However, the integration of these highly heterogeneous modalities inevitably introduces severe physical cross-talk, presenting a critical circuit-design challenge. For instance, simultaneous electrochemical sensing often requires macro-scale voltage sweeps (e.g., scanning from −0.2 V to +0.4 V for dopamine detection). Such massive voltage fluctuations can easily saturate adjacent high-gain, low-noise electrophysiological recording amplifiers, necessitating advanced time-division multiplexing or spatial decoupling strategies [103]. Similarly, integrating optogenetics with MEAs faces significant optical-electrical interference. High-intensity optogenetic illumination hitting the semiconductor substrate can generate photo-generated charge carriers—known as the Becquerel effect—resulting in massive photoelectric artifacts and baseline drifts that obscure true neural spikes. Mitigating these artifacts requires the implementation of highly opaque metal shielding layers or the development of fully transparent, wide-bandgap electrode materials [106].

## 6. Applications of MEAs for Brain-on-a-Chip

The above-mentioned various MEAs, with their on-chip amplification, high-density parallel recording, and closed-loop feedback capabilities, have become the technological cornerstone for brain-on-chip systems, particularly in biological computing and network plasticity research.

In biological computing, Cortical Labs built “DishBrain” using 1024-channel CMOS HD-MEAs, where cultured neurons learned to play Pong within 20 min under closed-loop feedback [2]; their CL1 platform later extended this to control the game Doom, suggesting that in vitro neural networks possess the foundational capacity for complex spatiotemporal information processing [107]. The Indiana University team integrated human brain organoids with a multi-channel active MEAs to construct the “Brainoware” reservoir computing system, using spatiotemporally encoded voltage pulses to achieve Japanese vowel recognition and chaotic system prediction [1].

In network plasticity and closed-loop control, Schröter et al. utilized 1024-channel CMOS HD-MEAs to continuously record brain organoids for up to 100 days, enabling single-cell-level tracking of axonal conduction velocity and network reorganization, which are key capabilities for understanding learning and plasticity in brain-on-chip models [108,109]. The Tianjin University team developed “MetaBOC” using active MEAs closed-loop control to enable real-time robot control by cultured neural networks, achieving obstacle avoidance, target tracking, and precision grasping [4].

Beyond advanced biological computing and network plasticity, traditional 2D passive MEAs continue to play an indispensable role in disease modeling and high-throughput drug screening. Due to their robust stability, cost-effectiveness, and compatibility with multi-well formats, 2D passive MEAs remain the gold standard for in vitro neurotoxicity assays. For instance, they are extensively utilized to evaluate the pharmacological effects of anti-epileptic drugs, screen environmental neurotoxins, and model neurodegenerative diseases using human induced pluripotent stem cell (hiPSC)-derived neuronal networks, offering a highly scalable platform for preclinical pharmacology [110,111].

Concurrently, the rapid emergence of 3D MEAs has significantly extended BOC applications to complex 3D organoids and multi-regional assembloids. By physically wrapping around or penetrating into the tissue, 3D MEAs capture the spatiotemporal dynamics of neurodevelopment and disease progression that are otherwise inaccessible to planar arrays. These interfaces have been pivotal in studying altered network bursting behaviors in organoid models of neurodevelopmental disorders (such as Rett syndrome) [112] and mapping long-range functional connectivity across fused brain regions, providing unprecedented insights into the functional architecture of 3D neural models [92].

It should be noted that currently, most of the above brain-on-chip applications rely on CMOS-based active MEAs. Although TFT-based MEAs offer unique advantages in large-area transparency and flexibility, their limited readout channels, inferior on-chip signal conditioning, and lower system integration have prevented large-scale adoption in brain-on-chip applications. However, with continued improvements in TFT-CMOS backend compatibility and the maturation of high-mobility oxide semiconductors like indium gallium zinc oxide (IGZO), TFT-based MEAs are expected to complement CMOS in emerging directions such as large-field-of-view imaging, flexible conformal interfaces, and degradable systems.

In order to clarify the attributes and representatives of various MEAs used in BOC today, we have extracted some representative works mentioned in this article and summarized them in Table 10.

In addition, to synthesize the engineering compromises discussed across the preceding sections and provide a practical selection guide for BOC researchers, Table 11 summarizes the key advantages, primary trade-offs, and ideal application scenarios for the distinct MEA architectures.

## 7. Conclusions and Future Perspectives

Microelectrode array (MEA) technologies for BOC applications have undergone a transformative evolution. The field has successfully advanced from traditional passive planar electrodes to highly integrated active CMOS/TFT arrays, and further towards tissue-conformal 3D architectures. By effectively circumventing the mechanical mismatch inherent to rigid probes, modern 3D MEAs—through both endogenous integration and exogenous wrapping strategies—enable non-destructive, long-term bidirectional communication with complex brain organoids.

Nevertheless, to fully transition from proof-of-concept demonstrations to standardized bio-computing platforms, several critical technical bottlenecks must be addressed. First, the chronic stability of ultra-flexible MEAs remains severely limited; material degradation and encapsulation delamination in warm, aqueous culture environments necessitate breakthroughs in nanoscale hermetic coatings (e.g., atomic layer deposition) [96] and robust carbon-based nanocomposites [97,98]. Second, as channel counts scale to the tens of thousands to achieve single-cell resolution across macroscopic tissues, managing data bandwidth, thermal dissipation, and power consumption has become a severe hardware constraint [117]. Third, physical cross-talk in multi-modal systems—where electrochemical voltage sweeps or high-intensity optogenetic illumination interfere with adjacent high-gain electrophysiological amplifiers—requires innovative circuit-level decoupling and advanced substrate engineering [106].

Looking forward, overcoming these hardware limits heavily relies on the convergence of MEAs with advanced microelectronics and targeted machine learning algorithms. Rather than a vague conceptual integration, edge-AI and neuromorphic computing are increasingly required to resolve specific hardware bottlenecks in BOC systems, such as real-time spike sorting from massive high-density arrays, data bandwidth reduction, and zero-latency closed-loop stimulation control [118,119]. Ultimately, replacing overly ambitious expectations with these concrete, AI-driven hardware solutions and next-generation compliant materials will steadily advance the foundational research of organoid intelligence and highly integrated in vitro disease modeling.

## Figures and Tables

**Table 1:** Summary of representative works in BOC in recent years.

Representative BOC Works	Structure or Principles	Application	Significance	Ref.
DishBrain system by Cortical Labs in 2022	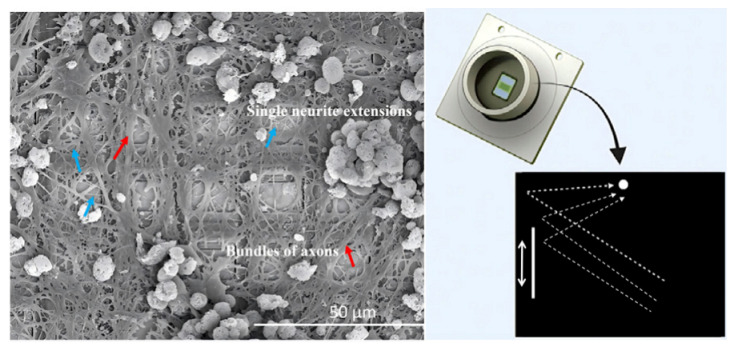 HD-MEA covering cells Control Pong game	Control the hiPSC neurons on chips to play the Pong game	The first real-time synthetic biological intelligence platform.	[2]
The Brain in a Box system by the University of Illinois in 2023	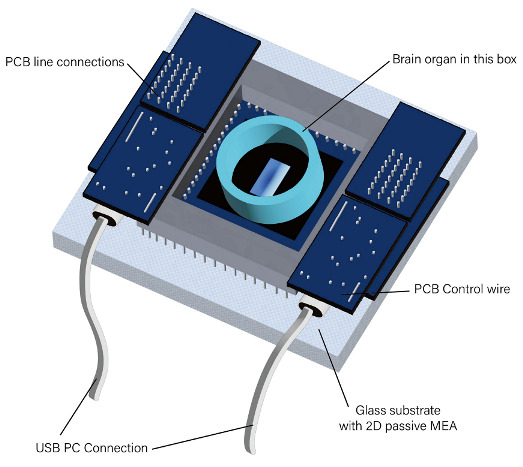 MEA system and overall structure	Enable neurons and the chip to collaborate to do signal recognition and processing tasks	The prototype of realizing the intelligent integration of carbon-based & silicon-based biology.	[6]
Brainoware by Indiana University in 2023	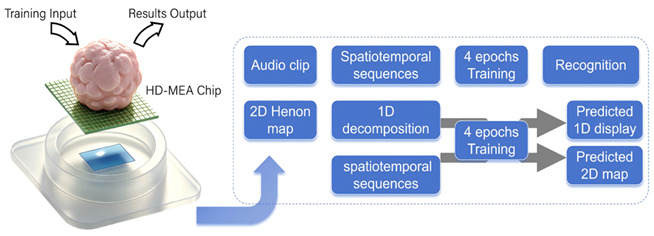 System and methods structure	Speech recognition classification task Use the brain to predict a nonlinear chaotic equation	Enable human brain organoids to self-organize into functional neural networks (ONNs) for brain-inspired AI hardware.	[1]
Neuroplatform by FinalSpark in 2024	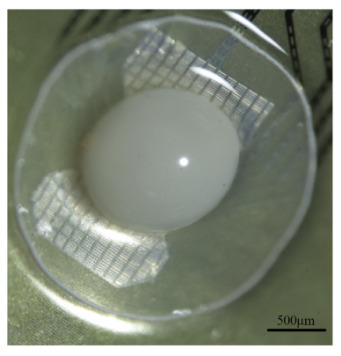 MEA covering cells 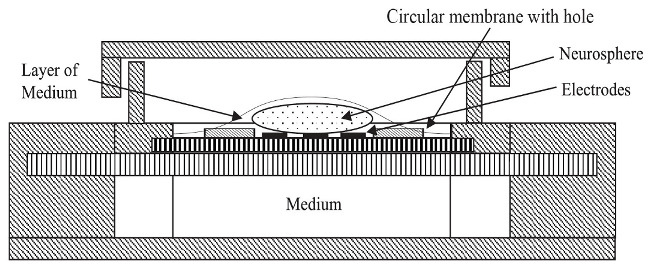 Overall structure	Ultra-low power consumption Able to collect over 18 TB of data Conduct research using interactive computing API can control pumps to release the molecules for complex experiments	The first interactive computing biological processor.	[7]

The figures (DishBrain & Neuroplatform) cited in this table were reprinted with permission from Creative Commons CC-BY license, and the figures (Brainoware & the Brain in a Box) were redrawn by the authors based on the reference [1,6].

**Table 2:** Summary of typical hardware core evaluation dimensions for MEAs.

Evaluation Dimension	Core Metrics	Specific Parameter Specifications	Ref.
Signal Acquisition & Recording	Target Signal Range Sampling Rate Input-Referred Noise Electrode Size Electrochemical Impedance	EAPs (Extracellular Action Potentials): Amplitude 10–500 μV(LFPs) (Local Field Potentials): 0.5–300 Hz, Amplitude 0.1–5 mV ≥10–20 kHz <5 μVrms (0.1 Hz–10 kHz) Micron-sized (10–50 μm in diameter) 100 kΩ–1 MΩ (at 1 kHz)	[8,9,10,11,12]
Precise Stimulation & Interaction	Charge Injection Capacity Safe Electrochemical Window	data > 0.5–1 mC/cm^2^ −0.6 V to +0.8 V (vs. Ag/AgCl, to avoid irreversible faradaic reactions)	[11,13]
Biocompatibility & Stability	Long-Term Culture Lifespan Cell Viability Inflammation Control Environmental Stability	Supports continuous culture for weeks to months (typically 4–52 weeks) >90% (compliant with ISO 10993-5 standards) No significant release of inflammatory cytokines (e.g., IL-6, TNF-α) Electrochemically stable in cell culture media (37 °C, pH 7.4) with minimal dissolution rates	[14,15,16,17]

**Table 3:** Electrochemical properties of typical electrode materials.

Material Type		CIC/CSC	Impedance@1 kHz	Feature	Ref.
Metal	Pt	0.05~0.3 mC/cm^2^	50 kΩ~1 MΩ	Good biocompatibility, low CIC	[11]
	Au	<0.1 mC/cm^2^	383.76 ± 31.46 kΩ	Chemically inert, high interface resistance	[35]
Improve metal	IrOx	3~5 mC/cm^2^ (CSC)	~4.5 kΩ	High charge storage capacity, good stability	[37]
	TiN	0.87 mC/cm^2^ (CIL)	~52.3 kΩ	High stability	
Conductive polymer	PEDOT: PSS	5~15 mC/cm^2^	68.9 ± 4.39 kΩ@diameter 10 μm/ 20.0 ± 1.89 kΩ@diameter 30 μm	Good flexibility, excellent biocompatibility	[34,35]
	PPy	2~10 mC/cm^2^	15.7 ± 1.46 kΩ	Good electrical conductivity, poor long-term stability	[34]
	PANI	1~5 mC/cm^2^	/	Simple synthesis, good environmental stability	[27]
Nanocomposites	PEDOT/CNT	1.21 mC/cm^2^	15.5 ± 1.19 kΩ	Good adhesion	[34]
	PEDOT-PSS-CNT	/	1.04 ± 0.17 kΩ	Low mechanical mismatch	[36]
	MWCNTs/PEDOT: PSS	/	36.41 ± 3.07 kΩ	Good SNR	[35]
Nanostructured metal	Pt grass	/	~1 kΩ	High surface area nanostructure	[22]
	Pt black	/	39.3 ± 6.36 kΩ	Traditional low-resistance coating, average mechanical stability	[38]
Carbon nanomaterials	Carbon nanotube (CNT) coating	1~10 mC/cm^2^	/	High specific surface area, high mechanical strength	[32]

**Table 4:** Summary of two typical structure MEAs.

Typical Structure	Switch Matrix (SM)	Active-Pixel Sensor (APS)
Principle	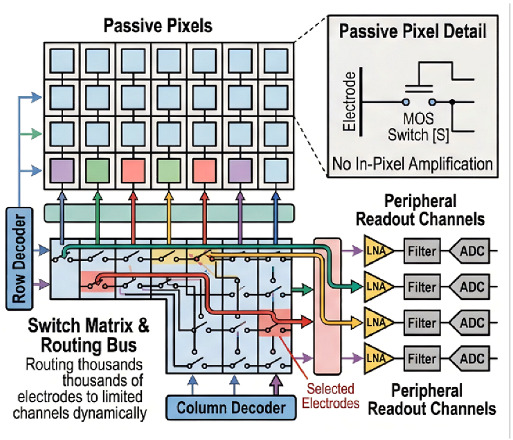	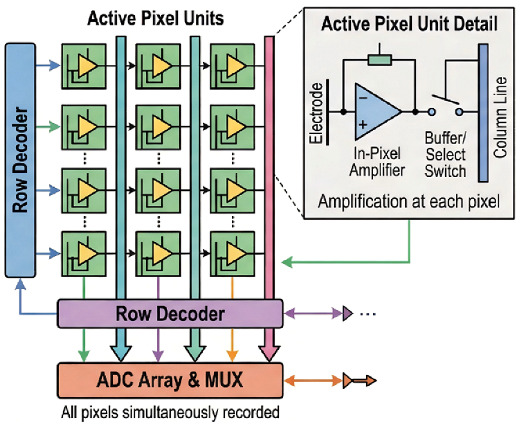

**Table 5:** Summary of representative CMOS-based active MEAs.

Architecture Type	Representative Work	No. of Electrodes	No. of Channels	Electrode Spacing (μm)	Noise (uVrms)	Spike Amplitude (μV)	Sampling Rate (kHz)	Biocompatibility	Ref.
Switch Matrix (SM)	Fery 2010	11,016	126	17.8	2.4	42~208	20	/	[43]
	Ballini M 2014	26,400	1024	17.5	2.4 (AP) 1.8 (500 Hz~3 kHz)	42~208	20	>5 months	[45]
	Vijay V 2016	59,760	2048	13.5	/	/	/	/	[46]
	Yuan 2020	59,760	4096	13.5	/	/	/	/	[47]
Active-Pixel Sensor (APS)	Berdondini 2009	4096	4096	42	/	/	7.8	/	[52]
	Sony 2018	Scalable to 6912	Scalable to 6912	12 (potential)	4.8	21~104	/	/	[54]
	IMEC 2019	16,384	1024	15	/	144.8	/	Long-time	[48]
Dual-mode	Yuan 2018	19,584	19,584 (APS) 246 (SM)	18	APS: 10.9 SM: 3.1	APS: 9~46 SM: 32~161	APS: 11.6 SM: 24.4	/	[56]
	Southeast University 2025	24,576	24,576 (Electrical) 24 (Chemical)4	18	Electrical: 2 fA/√Hz Chemical: /	/	Electrical: 5 Chemical: 70	Primary neuron culture	[57]
Vertical Nanoelectrode	Abbott 2017	>1000	>1000	/	Intracellular SNR > 100× improvement	Extracellular <5 Intracellular >500	/	235 cardiomyocytes synchronized	[58]
Sub-array multiplexing	-H. Cha 2022	24,320	380	/	/	/	/	/	[55]

**Table 6:** Summary of representative TFT-based active MEAs.

Solution	Representative Work	Electrodes	Pitch (μm)	Typical Impedance	SNR/Noise	Bandwidth/Cut-off Frequency	Optical Transmittance	Substrate Material	Ref.
Transparent TFT Hybrid Array	Shaik 2020	30,000	50–100	0.4 MΩ~2 MΩ	SNR: 80 dB	~2 kHz (3 dB)	>90% (visible light)	Glass	[59]
Large-Area TFT Array	Hu 2025	22,500	100	0.4 MΩ~2 MΩ	/	~7 kHz	ITO transparent	Glass	[60]
Flexible TFT Multiplexed System	Sheng 2025 (NeuroCam)	4096	150	10 kΩ~10 MΩ	Static: 2.3 ± 0.3 μVrms Dynamic: 63.6 ± 24.1 μVrms (4096 ch)	>1 kHz (3 dB)	Transparent	Polyimide (flexible)	[61]

**Table 7:** Quantitative engineering comparison between CMOS and TFT MEA platforms.

Engineering Metrics	CMOS-Based MEAs (e.g., Silicon)	TFT-Based MEAs (e.g., IGZO, Organics)	Critical Implications for BOC Applications
Carrier Mobility (Root Cause)	Ultra-high (~10^3^ cm^2^/V·s)	Low to moderate (<20 cm^2^/V·s for IGZO; <1 cm^2^/V·s for organics)	Low mobility in TFTs is the fundamental bottleneck limiting switching speed and amplification efficiency.
Channel Density	Massive (>10,000 channels/mm^2^)	Low to moderate (<1000 channels/mm^2^)	CMOS achieves single-cell/sub-cellular resolution; TFT is currently limited to macro-regional network mapping.
Dynamic Noise Floor	Ultra-low (<5 μV rms )	Moderate to high (~30–60 μV rms)	CMOS readily resolves subtle microvolt LFPs; TFT intrinsic noise often obscures weak synaptic activities.
Max Sampling Rate/Ch	High (>20 kHz to MHz range)	Limited (<10 kHz, typically 1–5 kHz)	CMOS fully captures fast-spiking action potentials; TFT is prone to signal aliasing for high-frequency spikes.
Drive Voltage & Power	Low logic voltage (1.2–3.3 V), ~μW per channel	High drive voltage (>5 V up to 15 V), leading to higher localized power	High voltages in TFTs increase Joule heating risk, which is highly detrimental to temperature-sensitive living organoids.
Substrate Mechanics	Rigid (Silicon die)	Rigid (glass substrate in most cases), flexible (Polyimide, Parylene, etc.)	CMOS inherently causes mechanical mismatch; TFT uniquely enables 3D conformal wrapping and dynamic stretching.
Fabrication & Cost	Standard CMOS foundry processes (High prototyping cost, extreme lithography limits)	Low-temperature processing (High compatibility with polymers, large-area scalability)	TFT is ideally suited for scalable, cost-effective, large-area disposable culture multi-well plates.

**Table 8:** Summary of mechanical and electrical properties of common flexible substrate materials for BOC applications.

Material	Young’s Modulus	Tensile Strength	Dielectric Constant	Resistivity/Conductivity	Refs.
PI	4.4 GPa	>100 MPa	2.84 @ 1 MHz	Insulation	[71]
PDMS	0.5~3 MPa	2–10 MPa	2.7–3.0 @ 1 kHz	Insulation	[72,73]
Parylene-C	2.4~4.0 GPa	50–100 MPa	3.1–3.2 @ 1 kHz	Insulation (>10^16^ Ω·cm)	[74]
SU-8	2.92~3.48 GPa	50–100 MPa	3.5–4.0 @ 1 kHz	Insulation (>10^15^ Ω·cm)	[75]
TPU	10~100 MPa	10–50 MPa	4.0–6.0 @ 1 kHz	Insulation (>10^12^ Ω·cm)	[76]

**Table 9:** Summary of typical structure and features of MEAs Integration with Multi-Functional Modules.

Representative Integrated Works	Typical Structure	Advantages	Application scenarios
MEAs Integration with Microfluidics and Flexible Interfaces	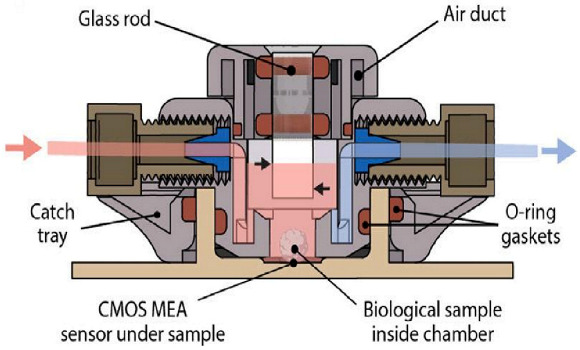 Voitiuk 2025 [99]	Integrated microfluidics, HD-MEA and microscopy imaging to support multi-modal simultaneous monitoring Fully automated organoid culture and data collection Low contamination risk and batch-to-batch variability Modular, low cost, easy to expand Medium replacement operation without interruption	Long-term brain organoid culture and neurodevelopmental research Dynamic studies of neurological disease models Cross-laboratory collaboration, shared research and remote teaching Enhance traditional neural research with MEAs in BOC
MEAs Integration with Optoelectronic Modules	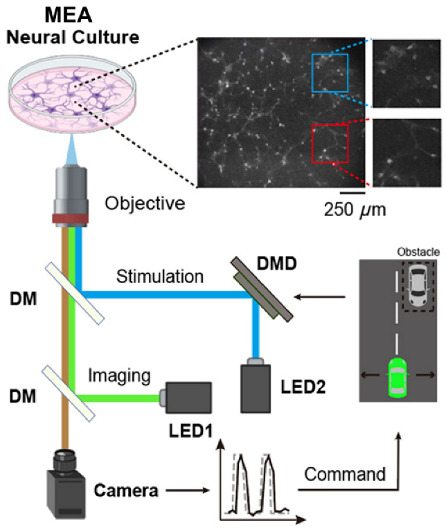 Deng 2025 [102]	All-optical method achieves high spatial and temporal resolution control and recording High computational efficiency Improve stimulation efficiency and biosafety Optogenetic stimulation significantly enhances network properties	Neural-robot control (e.g., virtual/real robot obstacle avoidance navigation) Brain–computer interface and biological hybrid system Research on neural plasticity and optogenetic regulation Low-power brain-inspired computing and physical reservoir computing Optogenetic intervention in neurological disease models and drug screening
MEAs Integration with Electrochemical Sensors	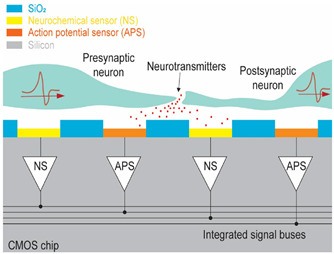 Mulberry 2023 [103]	Simultaneous recording of electrophysiological (action potential) and electrochemical (neurotransmitter release) signals Compact design Easy to form a staggered checkerboard array with high spatial resolution Enables more precise capture of neural signals that rely on the dynamic release of neurochemical transmitters	Analyze the causal relationship between dopamine, glutamate, acetylcholine and other transmitters and specific discharge patterns Research on the interaction mechanism between transmitter release disorders and abnormal discharges under pathological conditions Drug screening and neurotoxicity assessment
MEAs integration with 3D Scaffolds	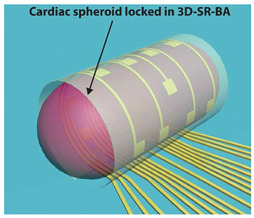 Kalmykov 2019 [105]	Full surface, multi-site simultaneous recording Supports both passive sensor and active sensor modes The mechanical structure of the scaffold can be flexibly adjusted to adapt to 3D cell spheroids of different sizes Excellent biocompatibility due to its soft texture and other characteristics	3D monitoring of the effects of drugs on electrical signal conduction in cardiomyocyte spheres to assess the risk of arrhythmia Construction of a spatiotemporal map of the field and action potential propagation in space Tissue engineering and regenerative medicine

The figures cited in this table were all reprinted with permission from Creative Commons CC-BY license.

**Table 10:** Summary of representative MEA technologies for Brain-on-a-Chip applications.

Technology Category	Representative Work	Array Scale&Spatial Resolution (Pitch/Size)	Key Hardware Metrics (Impedance/Noise/Stability)	Substrate & Material	Core Features	Ref.
2D Passive MEAs	Commercial (e.g., MCS MEAs2100)	60–256 ch; Pitch: 100–200 μm	Impedance: 50 kΩ~1 MΩ; Stable for weeks	Glass; TiN electrodes	Highly transparent substrate	[113]
	Vertical Nanopillars (Xie 2012)	Ch: /; Pitch: 2 μm	Extracellular to intracellular	Silicon-based	High-SNR intracellular recording	[42]
	Transparent Nanotubes (Lee 2024)	High density	High SNR intracellular extraction	Single-layer graphene; ZnO nanotubes	Fully transparent architecture	[41]
2D Active CMOS-MEAs	Switch-Matrix (Ballini 2014/MaxOne)	26,400 electrodes; 1024 ch; Pitch: 17.5 μm	Noise: 2.4 μVrms; Pt-Black drops impedance to 2.0 nF	Single-crystal Silicon; Pt/Pt-Black	High spatial resolution (3265 sites/mm^2^)	[45]
	Active-Pixel Sensor (Berdondini 2009)	4096 electrodes; Pitch: 42 μm	Sampling: 7.8 kHz full-frame	Silicon	Simultaneous full-array recording	[52]
	Dual-Mode Chemical-Electrical (Xu 2025)	24,576 electrodes; 10 μm diameter; Pitch: 18 μm	Current noise: 2 fA/√Hz; 123 dB dynamic range	Silicon	In situ parallel monitoring	[57]
2D Active TFT-MEAs	Transparent Hybrid Array (Shaik 2020)	30,000 electrodes; Pitch: 50–100 μm	Impedance spectroscopy compatible	Glass; ITO transparent electrodes	Ultra-large area	[59]
	Flexible TFT (Sheng 2025/NeuroCam)	4096 ch; Pitch: 150 μm (based on typical scale)	Dynamic noise: 63.6 μVrms (4096 ch)	15 μm ultra-thin polymer (Polyimide)	Flexible-substrate active multiplexing; Conformal tissue attachment	[114]
3D Flexible MEAs	Mesh Electronics (Lieber)	High porosity (>80%)	/	SU-8 polymer scaffold	Endogenous integration; Seamless organoid mapping	[77]
	Kirigami Electronics (Rogers/Yang 2024)	/	Recorded spontaneous activity up to 179 days	Elastomers/Metals	Buckling-mediated exogenous wrapping; Basket-structure cortical envelopment	[80]
	e-Flower Platform (Lacour/Martinelli)	/	SNR outperforms planar 2D	Polyimide driven by polyacrylic acid hydrogel	Hydrogel-swelling-driven self-folding; Omnidirectional surface recording	[88]
	Intrinsically Stretchable (Bao/NeuroString)	/	Stretchability > 100%	PDMS/TPU elastomers	Soft-brain-mimicking modulus	[115,116]

**Table 11:** Comparative analysis of advantages, trade-offs, and ideal application scenarios for various MEA architectures.

MEA Architecture	Key Advantages	Primary Trade-offs & Limitations	Ideal Application Scenarios
Planar Passive MEAs	Robust, cost-effective, and easy to fabricate; highly standardized.	Wiring bottleneck restricts channel density; low spatial resolution.	High-throughput neurotoxicity screening; basic pharmacological assays.
Planar Active CMOS-MEAs	Ultra-high density (sub-cellular resolution); ultra-low noise floor; on-chip signal processing.	A rigid and opaque silicon substrate severely limits optical microscopy and optogenetics.	High-resolution spatiotemporal mapping; axon conduction velocity tracking; biological computing.
Planar Active TFT-MEAs	Large-area scalability; excellent optical transparency; mechanically flexible.	Higher dynamic noise floor; limited sampling rate/bandwidth due to low carrier mobility.	Simultaneous large-field optical imaging (calcium/voltage) and electrical recording.
3D Wrapping/Shell MEAs (Exogenous)	Allows on-demand interfacing with mature organoids; less invasive and potentially removable.	Primarily restricted to surface (cortical) electrical mapping; lacks deep-tissue penetration.	Drug testing on mature organoids; multi-regional assembloid interfacing.

## Data Availability

Data sharing is not applicable.
